# Mechanisms and situational patterns of anterior cruciate ligament injuries in professional women's football (soccer): A systematic video analysis across 25 leagues (2022–2024)

**DOI:** 10.1002/ksa.70528

**Published:** 2026-07-13

**Authors:** Wyatt Hampstead, Ric Lovell, Matthew Whalan, Lionel Chia, Joshua Bonney, Bradley Wakefield, Vanessa Crespin, Evangelos Pappas

**Affiliations:** ^1^ School of Medical and Indigenous Health Sciences University of Wollongong Wollongong New South Wales Australia; ^2^ Football Australia Sydney Australia; ^3^ La Trobe Sports and Exercise Medicine Research Centre La Trobe University Sydney Australia; ^4^ Cleveland Guardians Baseball Co Cleveland Ohio USA; ^5^ School of Health Sciences, Faculty of Medicine and Health The University of Sydney Sydney New South Wales Australia; ^6^ PREP Health & Performance Fairy Meadow Australia; ^7^ School of Mathematics and Applied Statistics University of Wollongong Wollongong Australia; ^8^ School of Health and Biomedical Sciences Royal Melbourne Institute of Technology Melbourne Australia; ^9^ Shanghai University of Medicine and Health Sciences Shanghai China; ^10^ Independent Researcher and Data Analyst

**Keywords:** ACL, biomechanics, fatigue, video analysis, women's football

## Abstract

**Purposes:**

To identify and describe the biomechanical characteristics and football‐specific contextual factors associated with anterior cruciate ligament (ACL) injuries in professional women's football.

**Methods:**

A media‐based prospective cross‐sectional study identified 247 ACL injuries across international matches and 25 national women's leagues (tiers 3–5) between 2022 and 2024 using a validated media‐based surveillance approach. Video footage was available for 127 injuries and analysed by three reviewers using Quality Appraisal for Sports Injury Video Analysis Study (QA‐SIVA) informed protocols and frame‐by‐frame analysis. Situational variables included player action (e.g., pressing, tackling, landing, kicking), contact mechanism, match status, positional role, playing surface, competition, pitch location and time of injury.

**Results:**

Most injuries (67%) occurred within the first 45 min and during defensive play (65%). Mechanisms were non‐contact (49%), indirect contact (41%) and direct contact (10%). Frequent scenarios included pressing/tackling (53%), being tackled (15%), regaining balance after kicking (7%) and landing from a jump (5%). Among the biomechanical subsample (57 frontal/transverse and 46 sagittal cases), commonly observed patterns during pressing or tackling included ~50° hip flexion, ~25° knee flexion, a neutral trunk position (~0°), hip abduction (66%) and knee valgus (54%).

**Conclusions:**

ACL injuries in professional women's football frequently occurred during defensive actions early in matches. Within the biomechanical subsample, these injuries commonly involved limited lower‐limb flexion and valgus knee alignment, although findings should be interpreted cautiously due to the limited sample size. Together, these findings provide descriptive insight into common injury scenarios in professional women's football and may help inform future prevention strategies and targeted biomechanical research.

**Level of Evidence:**

N/A.

**Abbreviations**
ACL: anterior cruciate ligament
FIICCS: Football Injury Inciting Circumstances Classification System
IC: initial contact
ICC: intraclass correlation coefficient
IF: injury frame

## INTRODUCTION

Anterior cruciate ligament (ACL) injuries are amongst the most severe and burdensome in football (soccer) [2], largely due to the extended recovery time and long‐term consequences for players. While they account for less than 3% of all football‐related injuries, they contribute to approximately 28% of total time‐loss, with rehabilitation often exceeding 9 months [25]. Return to play remains challenging, with women (female) players facing a re‐injury rate of up to 34% [4], and many are either retiring early [8, 21] or continuing to experience symptoms that affect performance (e.g., pain, weakness and psychological distress) [13].

Women footballers are disproportionately affected by ACL injuries [7]. Since the 1990s, studies have reported a two‐ to three‐fold higher risk in female players compared to their male counterparts [22, 46]. The underlying reasons are likely multifactorial, involving a complex interplay of intrinsic (physiology and anatomy) and extrinsic factors (playing surface, access to support resources, training load and fatigue) [37]. While some of these are non‐modifiable, others may be addressed through a better understanding of injury mechanisms and the specific contextual demands of the women's game [30, 37].

Video analysis has been employed to identify both situational and biomechanical characteristics surrounding ACL injuries [1, 12, 32]. Research indicates that 88% of ACL injuries in football are non‐contact or indirect contact in nature, often occurring during defensive actions such as tackling or pressing [1, 23, 32, 34]. These situations require rapid, high‐risk movements under pressure while tracking opponents or reacting to changes in play. Notably, ACL injuries in women's football frequently occur early in the match, with approximately 50% occurring before the 27th minute of playing time [26] and 41% of noncontact injuries within the first 15 min [1]. The early match period may represent a phase of heightened vulnerability due to elevated physical demands, which may promote acute or transient fatigue [17, 24]. Reduced neuromuscular readiness, when combined with the cognitive demands of rapid decision‐making under pressure, may contribute to suboptimal movement strategies and altered joint loading. These conditions are thought to increase mechanical stress on the knee and may elevate the risk of ACL injury, particularly during high‐intensity actions such as pressing or directional changes [40].

Despite increasing research interest in injury mechanisms within women's football, most of the current understanding of ACL injuries is still based on studies involving male athletes or a narrow sample of elite female teams. Women remain underrepresented in biomechanical video‐based studies, comprising less than 17% of total reported cases [12, 14, 18, 19, 20, 34, 39]. Existing studies have tended to focus on players from top‐tier leagues, which limits generalisability to the broader population of professional female footballers [1, 12, 32, 34]. Football‐specific contextual variables, such as match status, field location, type of contact or playing surface, are often not considered in detail in the available women's literature, despite their relevance to both injury risk and real‐world decision‐making. Recent frameworks, such as the Football Injury Inciting Circumstances Classification System (FIICCS), have attempted to standardise how these factors are captured and described [3]. Finally, there is limited application of validated quality frameworks such as Quality Appraisal for Sports Injury Video Analysis Study (QA‐SIVA), which are designed to improve methodological consistency and enhance the reliability of video‐based injury assessments by standardising definitions and ensuring inter‐rater agreement [28]. These limitations have contributed to a fragmented evidence base, and there remains a need for context‐specific, sex‐informed research to support more targeted approaches to injury prevention in women's football [37].

To address these limitations, this study aims to characterise the situational patterns, biomechanical features and contextual factors associated with ACL injuries in women's football. Insights generated from this analysis are intended to inform the future development of targeted prevention strategies and return‐to‐play protocols tailored to the demands of the women's game.

## METHODS

### Injury surveillance and study design

This prospective observational study examined ACL injuries sustained by professional women's football players competing in official matches between February 2022 and December 2024. Eligible players were classified as tier 3–5 according to the McKay et al. Participant Classification Framework [33], representing 25 professional leagues and international competitions (Supporting Information S1: Table 1).

A media‐based surveillance approach was employed to identify ACL injuries, offering broader coverage and data access than direct reporting from team medical staff [31]. This methodology, previously validated in sports injury research [26], included searches of public sources (e.g., club statements, X/Twitter, Soccerdonna) using multilingual keywords (e.g., ‘ACL’, ‘ligament croisé’) to identify confirmed cases. All entries were cross‐verified by two researchers, and only cases where ACL injury was independently confirmed by multiple independent sources were included. Because injury identification relied on publicly available information rather than clinical records, magnetic resonance imaging (MRI) confirmation and the presence of concomitant injuries (e.g., meniscal or collateral ligament injuries) could not be independently verified.

### Video acquisition and eligibility

Video footage was sourced for 131 identified injuries from publicly available media platforms (YouTube, FA Player, NWSL, FIFA+). Videos were eligible if they included at least one clear camera angle of the injury event. Footage of poor quality, limited visibility or with unclear injury moments were excluded (*n* = 4). The number of camera angles per injury video varied: 44% of cases were captured from one angle, 37% from two, 16.5% from three and 1.6% from four. Eligible footage was trimmed to show 10–15 s preceding and 5–10 s following the estimated injury frame (IF; TapRecord, Vidline Inc; [19]).

### Injury mechanism, situational classification and football contextual factors

Three reviewers (two sports scientists and one physiotherapist) with ≥5 years' experience in sports medicine and ACL injury management independently analysed the footage frame‐by‐frame using Kinovea (Version 1.20; https://www.kinovea.org). Assessment followed established video analysis frameworks [5, 19, 47], including field location (short/long axis), nature of play (defensive/offensive) and player contact type (non‐contact, indirect or direct) [5, 19]. Defensive pressing was defined as coordinated pressure to reduce time and space; tackling was defined as an individual action to dispossess an opponent [38]. Injury mechanisms were classified as non‐contact (no external contact prior to or during injury), indirect contact (contact occurred but not to the injured knee) or direct contact (force applied directly to the injured knee), following the definitions by Della Villa et al. [19]. Situational patterns were identified according to the player's action at the time of injury (e.g., pressing, landing, kicking) (Supporting Information S1: Table 2). These categories were not statistically compared due to their distinct biomechanical nature and contextual variability.

### Biomechanical assessment

Kinematic analysis focused on two timepoints: initial contact (IC), defined as the moment the injured limb first contacted the ground, and the IF, estimated to occur approximately 40 ms after IC [19, 29]. For indirect or non‐contact injuries, researchers assessed injured‐limb loading, the number of planted feet at IF and the player's approach velocity in both vertical and horizontal directions. Approach velocity to the injury event was classified as either zero, low, high or uncertain in accordance with established methodology [19, 47].

Biomechanical observations were conducted using a structured framework, adapted from previous video‐based analyses [1, 19, 32] (Supporting Information S1: Table 3). Joint positions were evaluated sequentially from the trunk to the ankle across the sagittal (flexion/extension), frontal (abduction/adduction) and transverse (internal/external rotation) planes. Sagittal‐plane angles for trunk, hip, knee and ankle flexion, as well as trunk tilt, were estimated using open‐access software (ImageJ; [35]) and approximated to the nearest 5°. Frontal plane positions were assessed visually at both IC and IF, with any obscured or unavailable angles recorded as ‘unsure’. Due to the limitation inherent to two‐dimensional (2D) footage, biomechanical observations should be interpreted as visual estimates rather than precise measurements.

Following independent assessments, reviewers convened for a consensus meeting to determine the primary injury mechanism and situational pattern. A fourth reviewer was designated to adjudicate disagreements if required [1, 19, 32]; however, full consensus was achieved. All video footage was publicly available, and the players were deidentified.

### Football contextual factors

Contextual variables were extracted using the FICCS framework [3]. Data included match status (winning, drawing, losing), player position (forward, midfielder, defender and goalkeeper), competition type (domestic leagues, international club competitions and national team fixtures), playing surface (artificial, grass or hybrid), match half and time of injury. Substitution records from Soccerdonna, Soccerway and Sofascore were used to estimate player field time and cross‐validated against the game clock in the footage (Supporting Information S1: Table 4). Playing surface was verified online and, where necessary, by contacting venues. Because individual and team‐level exposure data (e.g., minutes played by surface, match status or competition) were not available, contextual variables are reported as counts and proportions of injuries only and cannot be interpreted as incidence rates or relative risks.

### Ethical considerations

All video footage and contextual data analysed in this study were publicly available and featured identifiable individuals already visible in mainstream media coverage. No personal or confidential information was collected. As there was no interaction with athletes and no private data used, the study met the criteria for ethical exemption. This exemption was granted by the Human Research Ethics Committee (HREC reference:2024/316).

### Statistical analysis

This study employed a descriptive observational design to identify and characterise patterns of ACL injury in women's football using prospectively collected video and contextual data. Given the diversity and qualitative nature of many injury scenarios, statistical comparisons between biomechanical or situational subgroups were not performed. These categories represent distinct movement strategies that do not lend themselves to meaningful inferential comparison without compromising interpretability. Instead, emphasis was placed on identifying clinically and ecologically relevant patterns to guide future research and prevention strategies. Additionally, since pressing and tackling share similar defensive and biomechanical characteristics, these scenarios were combined for temporal analyses within the results.

Descriptive statistics were used to summarise all variables. Continuous data are reported as mean ± standard deviation (SD), or median and range, depending on distribution. Categorical variables are presented as frequencies and percentages. Associations between injury occurrence and selected contextual factors (e.g., turf type, match status) were assessed using chi‐squared (*χ* ^2^). Statistical significance was set a priori at *α* level = 0.05.

To assess consistency between reviewers, Fleiss kappa was used for categorical variables (e.g., injury mechanism), and intraclass correlation coefficients (ICCs) were calculated for ordinal or continuous variables (i.e., joint angles). The Fleiss kappa for injury classification was 0.71 (substantial agreement), while ICCs for IC and IF identification ranged from 0.92 to 0.99. ICCs for kinematic assessment were excellent (0.91–0.95), except trunk tilt and rotation, which were rated as good (0.75–0.90). All statistical analyses were performed using RStudio (Version 4.3.2) and Microsoft Office version 365.

## RESULTS

### Injury identification and match context

**Figure 1:**
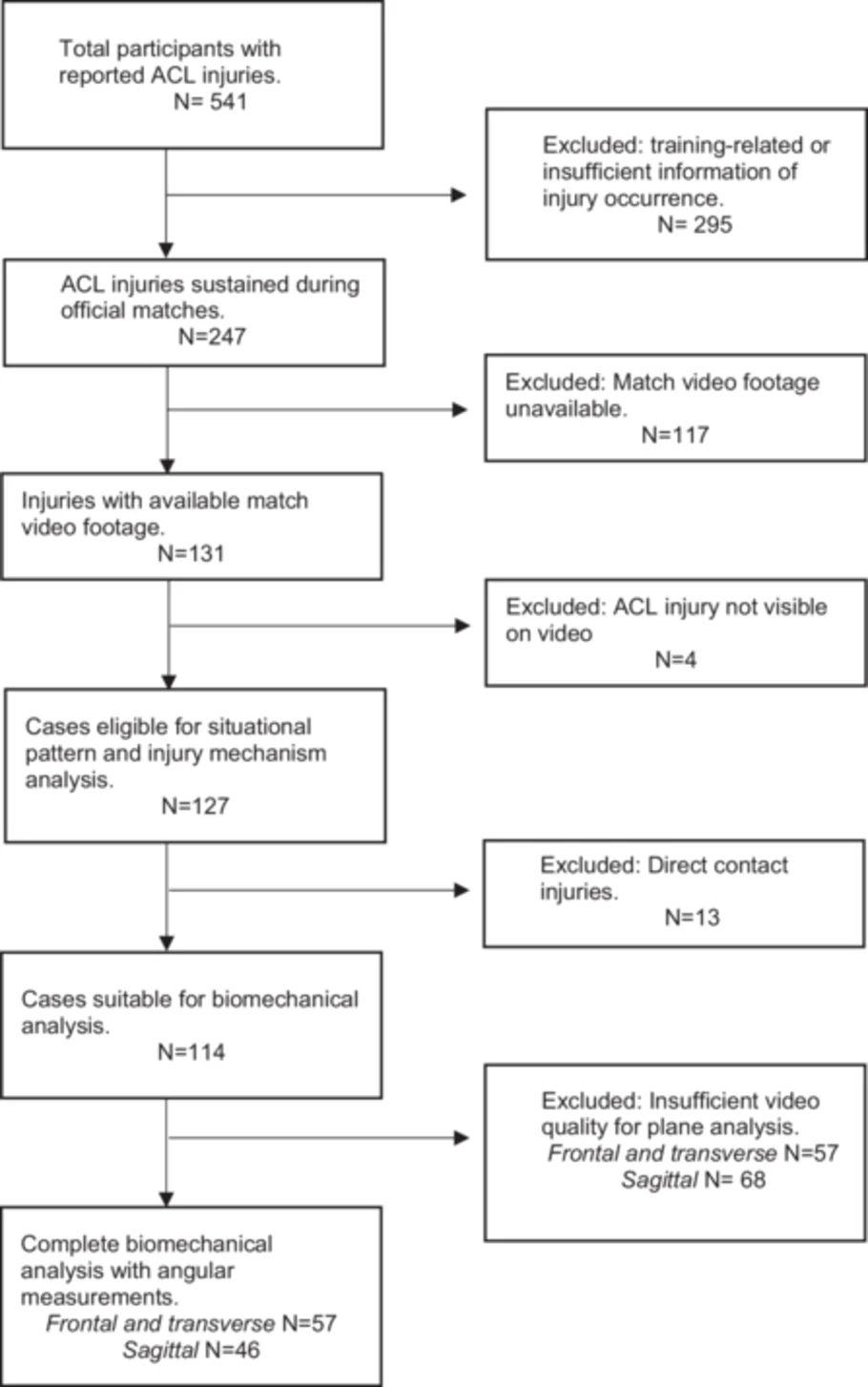
Flowchart of the study. ACL, anterior cruciate ligament.

Of the 541 ACL injuries identified (Figure 1), 247 occurred during official matches and were included in the analysis. Most occurred during domestic league matches (75.7%), followed by national team competitions (9.7%), domestic cup matches (7.7%), international club cups (2.4%), national team friendly matches (3.2%) and club friendly matches (0.8%).

Most match injuries occurred on natural grass (59.5%), followed by artificial turf (27.9%) and hybrid surfaces (8.1%). A greater proportion occurred when teams were drawing (44.9%), compared with losing (28.3%) or winning (23.5%). Starting players accounted for 90% of injuries. Midfielders were most frequently affected (36.8%), followed by defenders (30.0%), forwards (27.1%) and goalkeepers (6.1%). Of the 247 match injuries, 131 had associated video footage. Four videos were excluded due to inadequate quality or visibility, leaving 127 injuries for situational and injury mechanism analysis (median player age = 24 years).

### Injury mechanisms according to situational patterns

**Figure 2:**
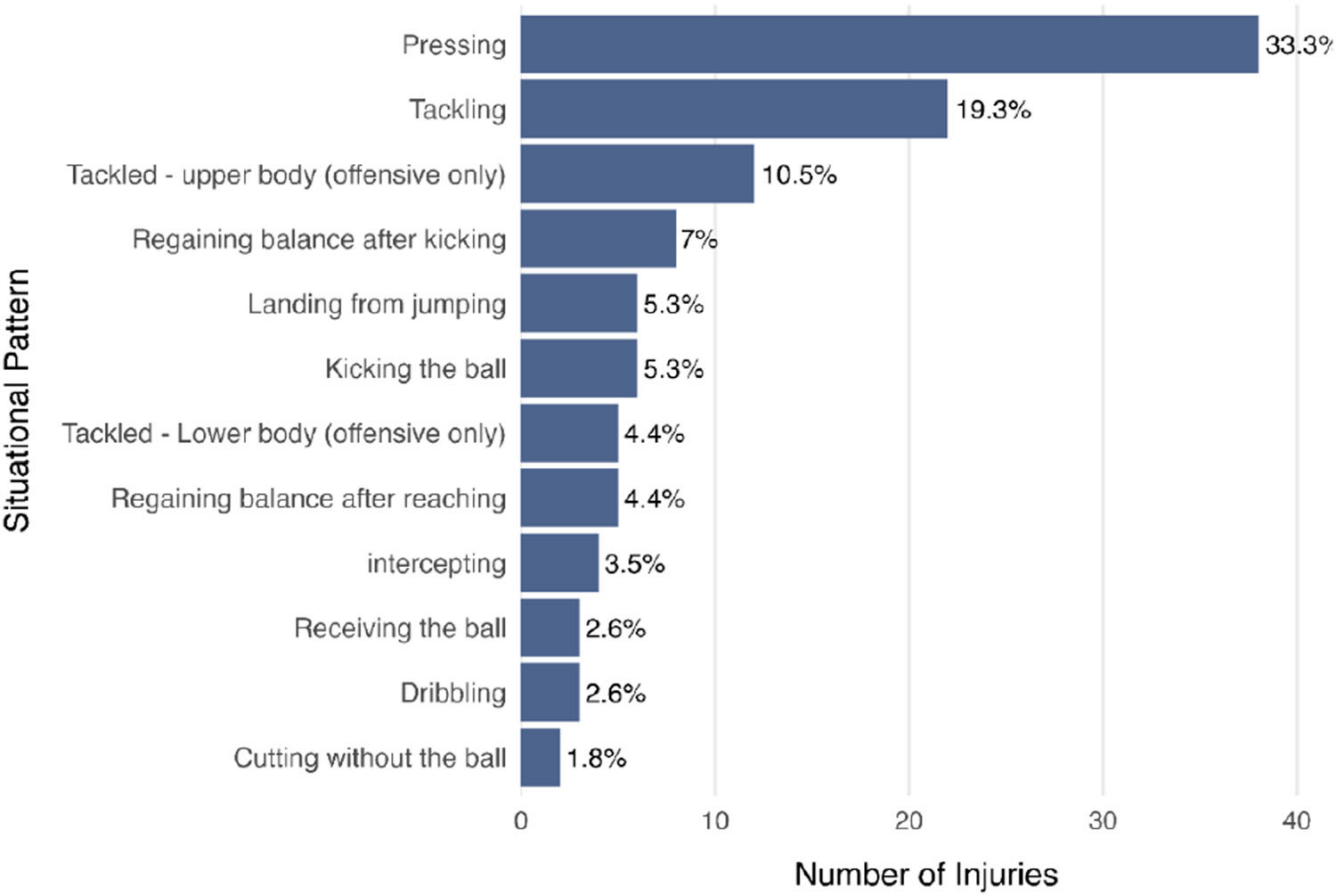
Distribution of situational patterns among indirect and non‐contact ACL injuries. ACL, anterior cruciate ligament.

Among the 127 video‐confirmed injuries (Table 1), 49% were classified as non‐contact, 41% as indirect contact and 10% as direct contact. The majority occurred during the defensive phase of play (65%). The most common injury scenarios were pressing (*n* = 38, 33.3%) and tackling (*n* = 22, 19.3%), followed by being tackled (13.7%), jump landing (5.3%) and regaining balance after reaching and kicking (4.4% each). Less frequent scenarios included intercepting, dribbling, receiving or cutting without the ball (collectively 24%) (Figure 2). Direct contact injuries (*n* = 13) involved visible contact to the injured leg and occurred relatively evenly between offensive (53.8%) and defensive (46.2%) phases. Midfielders accounted for nearly half (46.2%), followed by defenders (30.8%) and forwards (23.1%).

**Table 1:** Within‐game characteristics of all ACL injuries and for noncontact and indirect contact injuries across identified situational patterns.^a^

Variable	Noncontact and indirect contact situational patterns (*n* = 114)						
	All video injuries (*n* = 127)	Pressing/Tackling (*n* = 60)	Being tackled (*n* = 17)	Regaining balance after kicking (*n* = 8)	Landing from jumping (*n* = 6)	Other (*n* = 23)	Total (*n* = 114)
Playing phase							
Offence	45 (35%)	0 (0)	17 (100%)	6 (75%)	1 (17%)	14 (61%)	38 (33%)
Defence	82 (65%)	60 (100%)	0 (0)	2 (25%)	5 (83%)	9 (39%)	76 (67%)
If contact where							
Upper body	42 (82%)	17 (85%)	10 (67%)	4 (100%)	3 (100%)	6 (100%)	40 (83%)
Pelvis	5 (10%)	2 (10%)	3 (20%)	0 (0)	0 (0)	0 (0)	5 (10%)
Uninjured leg	2 (16%)	1 (5%)	2 (13%)	0 (0)	0 (0)	0 (0)	2 (4%)
Injured leg	2 (2%)	0 (0)	1 (7%)	0 (0)	0 (0)	0 (0)	1 (2%)
Player contact at IF							
No	64 (50%)	35 (58%)	2 (12%)	5 (62%)	4 (67%)	17 (74%)	63 (55%
Yes	61 (48%)	25 (42%)	15 (82%)	3 (38%)	1 (17%)	6 (26%)	51 (45%)
If indirect contact where							
Upper body	37 (71%)	17 (65%)	10 (67%)	2 (67%)	2 (100%)	5 (83%)	37 (71%)
Pelvis	8 (15%)	3 (12%)	3 (20%)	1 (33%)	0 (0)	1 (17%)	8 (15%)
Uninjured leg	8 (16%)	6 (23%)	2 (13%)	0 (0)	0 (0)	0 (0)	8 (16%)
Injury classification							
Non‐contact	62 (49%)	35 (58%)	2 (12%)	5 (62%)	4 (67%)	17 (74%)	62 (54%)
Indirect contact	52 (41%)	25 (42%)	14 (82%)	3 (38%)	1 (17%)	6 (26%)	52 (46%)
Contact	13 (10%)	0 (0)	0 (0)	0 (0)	0 (0)	0 (0)	0 (0)
How many feet on the ground							
One		42 (70%)	11 (65%)	4 (50%)	4 (67%)	12 (52%)	73 (64%)
Two		13 (22%)	3 (18%)	2 (25%)	2 (33%)	9 (39%)	29 (25%)
Unsure		5 (8%)	3 (18%)	2 (25%)	2 (9%)	2 (9%)	12 (11%)
Leg loading at IF							
Injured leg		57 (95%)	16 (94%)	7 (88%)	5 (83%)	21 (91%)	106 (93%)
Uninjured leg		0 (0)	0 (0)	1 (12%)	0 (0)		1 (1%)
Unsure		3 (5%)	1 (6%)	0 (0)	1 (17%)	2 (9%)	7 (6%)
Horizontal speed							
Zero		0 (0)	0 (0)	0 (0)	0 (0)	1 (4%)	1 (1%)
Low		18 (30%)	5 (29%)	1 (12%)	2 (33%)	17 (74%)	43 (38%)
High		42 (70%)	12 (71%)	7 (88%)	4 (67%)	5 (22%)	70 (61%)
Unsure		0 (0)	0 (0)	0 (0)	0 (0)	0 (0)	0 (0)
Vertical speed							
Zero		24 (40%)	3 (18%)	1 (12%)	0 (0)	5 (22%)	33 (29%)
Low		35 (58%)	14 (82%)	6 (75%)	1 (17%)	16 (70%)	72 (63%)
High		1 (2%)	0 (0)	1 (12%)	5 (83%)	2 (9%)	9 (8%)
Unsure		2 (3%)	0 (0)	0 (0)	0 (0)	1 (4%)	3 (3%)

Abbreviations: IC, initial contact; IF, injury frame.

^a^ Data are reported as *n* (%).

**Figure 3:**
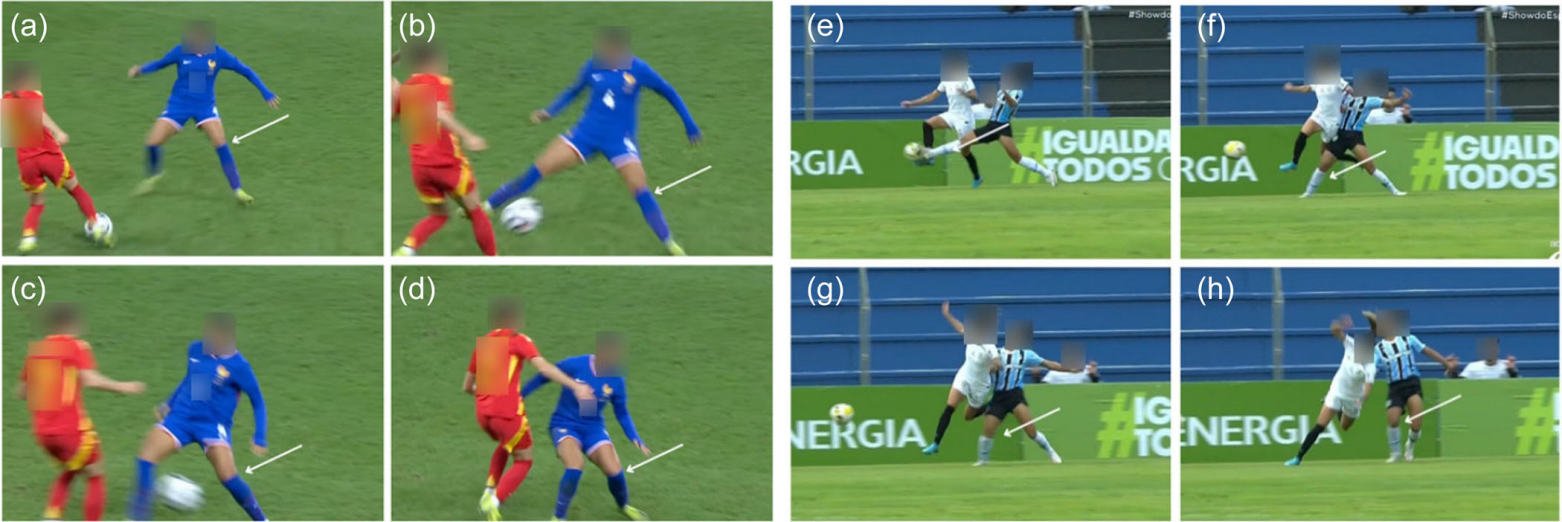
Examples of ACL injuries sustained during pressing and tackling. (a–d) Pressing injury involving the player in blue, with right ACL injury: (a) approaching the opponent, (b) initial contact, (c) injury frame, (d) loss of balance. (e–h) Tackling injury to the player in striped blue and black, with right ACL injury: (e) approaching the opponent, (f) initial contact and tackle, (g) injury frame and (h) loss of balance. ACL, anterior cruciate ligament.

The remaining 114 injuries were classified as either non‐contact or indirect contact. Injuries during pressing and tackling (*n* = 60) (Figure 3) occurred exclusively during defensive play and most frequently in the midfield third (54%) and right‐side corridor (41%) (Supporting Information S1). These injuries primarily affected defenders (45%), and most occurred without player contact before IF (65%) and were categorised as noncontact (58%). High horizontal speed was present in 70% of these cases, while vertical speed was low in 58%.

**Figure 4:**
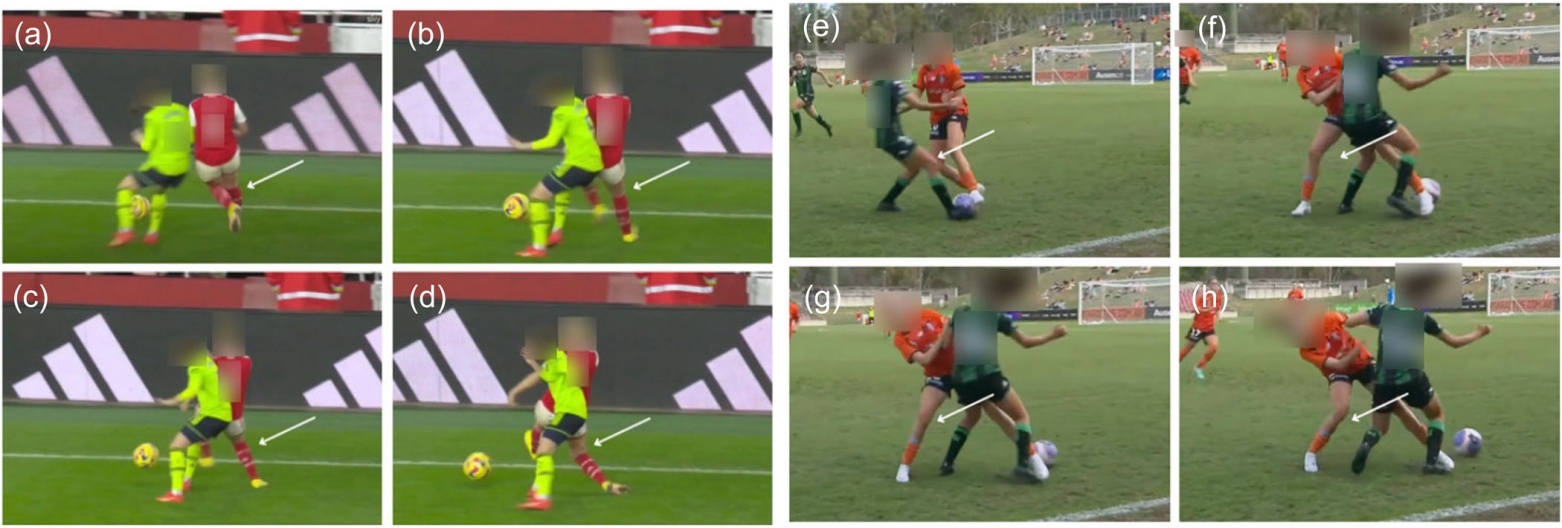
Examples of ACL injuries sustained while being tackled. (a–d) Injury to player in red and white, with right ACL injury following upper body contact: (a) mechanical perturbation, (b) initial contact of injured leg, (c) estimated injury frame, (d) loss of balance. (e–h) Injury to player in orange and white, with right ACL following lower limb contact: (e) mechanical perturbation, (f) initial contact of injured leg, (g) estimated injury frame and (h) loss of balance. ACL, anterior cruciate ligament.

Seventeen injuries occurred while players were being tackled (Figure 4). All occurred during offensive phases, most occurring in the midfield or offensive third (50% each), within the right‐side corridor (44%). The majority involved contact before IF (88%), often classified as indirect contact (82%) involving the upper body (73% at IF). Horizontal speed was high in 71% of these cases, while vertical speed remained low (82%).

**Figure 5:**
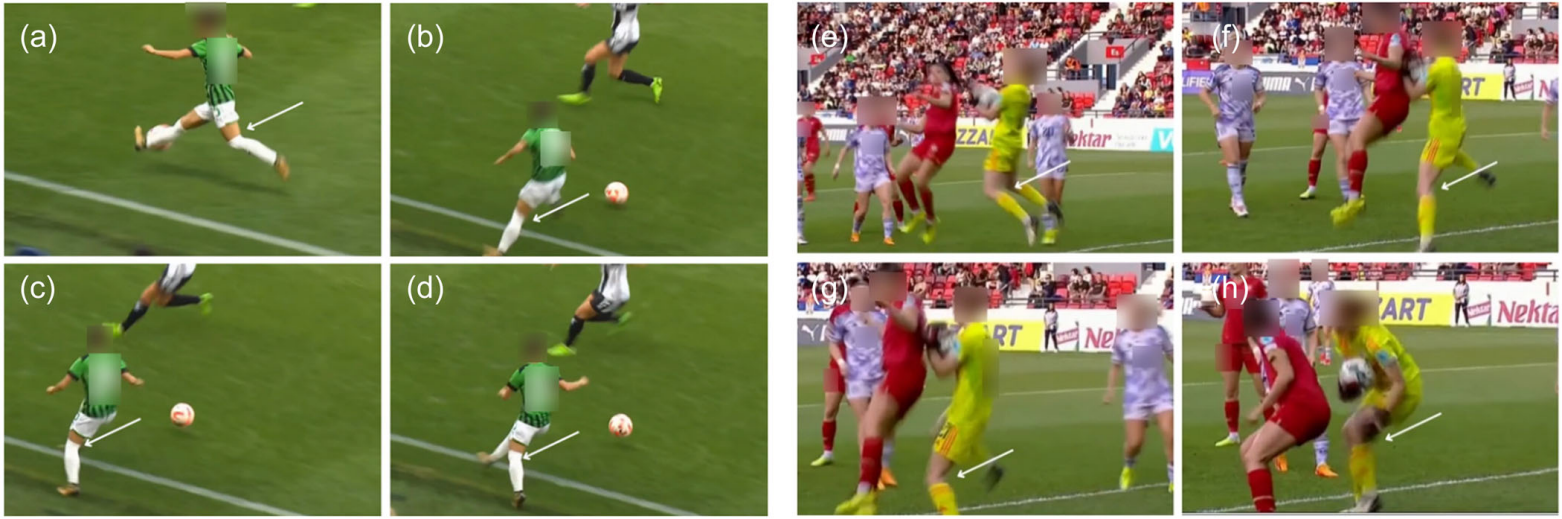
Examples of ACL injuries sustained while regaining balance after kicking and following landing from a high jump. Injury to player in green, with left ACL sustained while regaining balance after kicking: (a) kicking the ball, (b) initial ground contact, (c) estimated injury frame, (d) loss of balance. (e–h) Injury to the player in yellow, with left ACL injury sustained during landing from a jump. (e) Mid‐jump, (f) initial contact after landing, (g) estimated injury frame and (h) loss of balance. ACL, anterior cruciate ligament.

**Figure 6:**
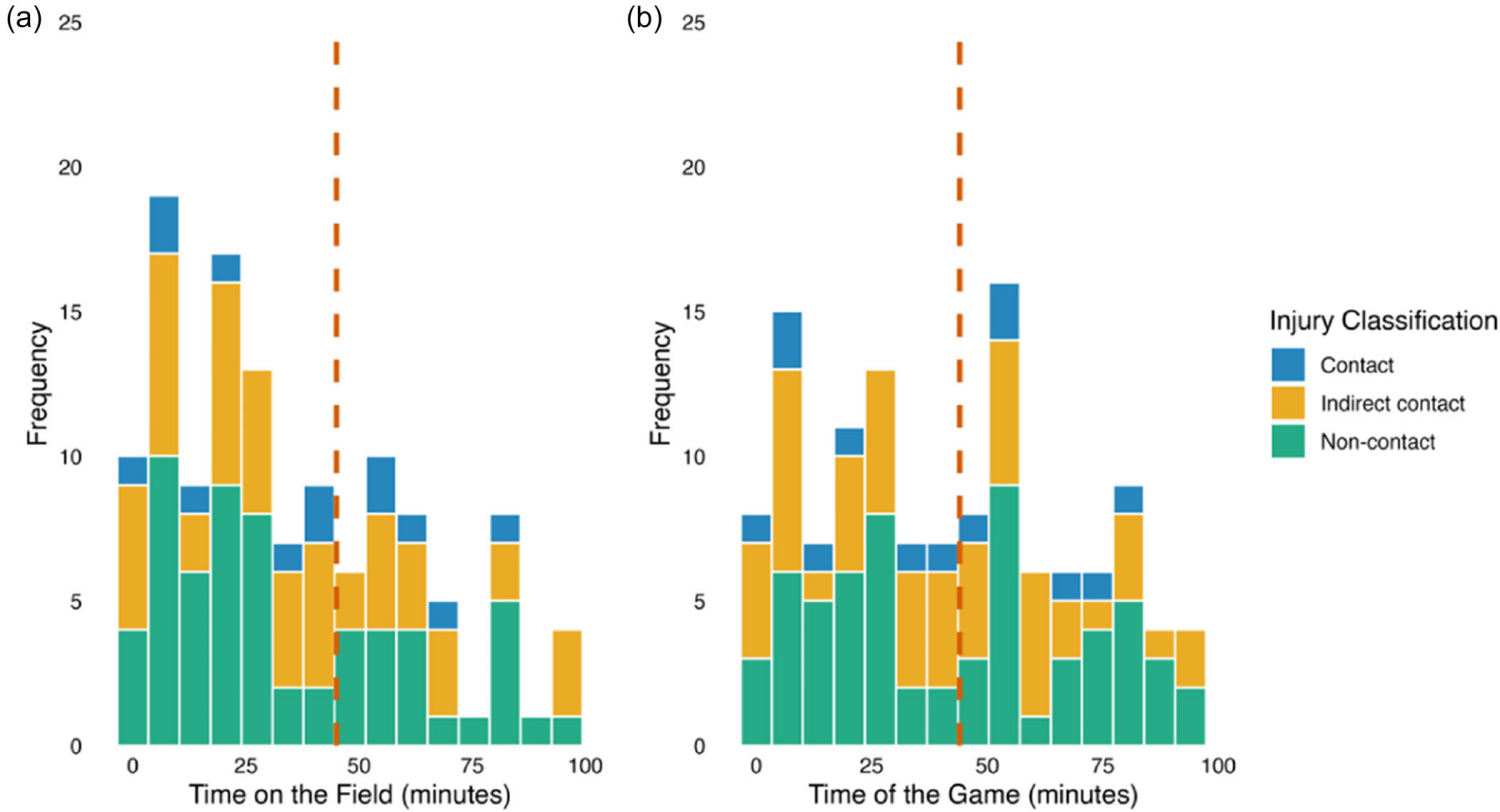
(a) Distribution of ACL injuries by time on the field (min) and injury classification. (b) Distribution of ACL injuries by time of the game (min) and injury classification. ACL, anterior cruciate ligament.

Injuries sustained while regaining balance after kicking (*n* = 8) were more common during offensive phases (75%), typically occurred in the offensive third (50%) and the middle corridor (62%), usually without preceding playing contact (62%). Horizontal speed was high in 88% of cases, and vertical speed remained low in 75% (Figure 5). Landing from a jump (*n* = 6) was predominantly associated with defensive actions (83%) and occurred most frequently in the defensive third (67%) and middle corridor (67%). Most injuries in this category were non‐contact (67%) and occurred at high horizontal (67%) and high vertical speed (83%) (Figure 6).

### Biomechanical analysis

Different situational patterns were associated with distinct sagittal plane (Table 2) biomechanical features (Table 2). During pressing and tackling, knee flexion increased from 24° at IC to 60° at IF, with flat foot positioning observed at IF in 69% of cases. Injuries sustained while being tackled showed greater trunk and hip flexion values, increasing from approximately 10° to 15° and 55° to 62.5°, and the highest incidence of toe strikes at IF (12%). Regaining balance after kicking was marked by trunk extension at IC (−10°), with relatively low knee flexion (15°). This pattern also demonstrated a notable increase in hip flexion (10°–30°) and ankle dorsiflexion (−25° to 0°). During landing from a jump, knee flexion at IC was approximately 40°, with similar increases in ankle dorsiflexion (approximately −20° to 5°) compared with the regaining‐balance pattern, while hip flexion remained similar between IC and IF (~30°).

**Table 2:** Sagittal plane values of non‐contact or indirect contact ACL injuries.^a^

Variable	Noncontact and indirect contact situational patterns					
	Total (*n* = 57)	Pressing/Tackling (*n* = 36)	Being tackled (*n* = 7)	Regaining balance after kicking (*n* = 5)	Landing from jumping (*n* = 3)	Other (*n* = 9)
Flexion angle (°) [+ flexion, − extension]						
Trunk at IC	0 (−15 to 30)	0 (−15 to 30)	10 (−10 to 25)	−10 (−10 to 10)	0 (−5 to 10)	−2.5 (−10 to 5)
Trunk at IF	5 (−10 to 25)	5 (−10 to 20)	15 (−5 to 25)	−5 (−10 to 15)	0 (0 to 5)	5 (−10 to 10)
Hip at IC	50 (10 to 90)	50 (10 to 70)	55 (40 to 90)	10 (10 to 50)	30 (15 to 30)	40 (10 to 70)
Hip at IF	55 (15 to 80)	55 (15 to 80)	62.5 (35 to 80)	30 (25 to 60)	30 (20 to 45)	50 (30 to 70)
Knee at IC	25 (0 to 75)	24 (0 to 65)	30 (5 to 75)	15 (10 to 30)	40 (20 to 45)	22.5 (5 to 60)
Knee at IF	55 (−20 to 90)	60 (−20 to 90)	50 (20 to 90)	45 (30 to 65)	60 (50 to 65)	50 (30 to 70)
Ankle at IC	−13 (−40 to 40)	−10 (−40 to 40)	−2.5 (−40 to 25)	−25 (−30 to −20)	−20 (−20 to 20)	−15 (−20 to 10)
Ankle at IF	0 (−25 to 40)	0 (−25 to 40)	−5 (−25 to 0)	0 (0 to 10)	5 (0 to 10)	−20 (−20 to 10)
Foot strike appearance at IC	*n* = 114	*n* = 60	*n* = 17	*n* = 8	*n* = 6	*n* = 23
Heel	32% (37)	22 (37%)	6 (35%)	3 (38%)	1 (17%)	6 (26%)
Flat	28% (32)	21 (35%)	3 (18%)	2 (25%)	2 (33%)	3 (13%)
Toe	0 (0)	0 (0)	0 (0)	0 (0)	0 (0)	0 (0)
Unsure	40% (45)	17 (28%)	8 (47%)	3 (38%)	3 (50%)	14 (61%)
Foot strike appearance at IF	*n* = 114	*n* = 60	*n* = 17	*n* = 8	*n* = 6	*n* = 23
Heel	0 (0)	0 (0)	0 (0)	0 (0)	0 (0)	0 (0)
Flat	58% (66)	41 (69%)	7 (41%)	5 (62%)	3 (50%	10 (43%)
Toe	4% (4)	2 (3%)	2 (12%)	0 (0)	0 (0)	0 (0)
Unsure	39% (44)	17 (28%)	8 (47%)	3 (38%)	3 (50%	13 (57%)

Abbreviations: 2D, two‐dimensional; ACL, anterior cruciate ligament; IC, initial contact; IF, injury frame.

^a^ Data are reported as median (range) visual estimates derived from 2D video footage and should be interpreted as approximate values rather than precise biomechanical measurements.

Frontal plane analysis (Table 3) demonstrated distinct movement patterns. During pressing and tackling, with 32%–37% of frontal plane observations classified as unsure, knee alignment appeared to shift from predominantly neutral at IC (55%) to more frequently valgus at IF (67%) among assessable cases. Hip internal rotation/adduction was observed in 63% of cases, accompanied by an increase in trunk rotation toward the uninjured side from 38% at IC to 65% at IF. Being tackled showed greater trunk tilt, increasing from approximately 15° at IC to 35° at IF. During regaining balance after kicking, trunk tilt decreased slightly (−5°), while knee valgus alignment increased (0%–38%), although no instances of valgus collapse were observed. For landing from jumping, trunk rotation toward the uninjured side increased (33%–67%), with modest increases in knee valgus (0%–17%) and no observed valgus collapse. Several variables showed substantial uncertainty. Overall, knee alignment was classified as unsure in 42%–46% of cases and foot position in 42%–46% of cases, with uncertainty reaching 65%–70% in some situational subgroups; therefore, these findings should be interpreted cautiously.

**Table 3:** Frontal and transverse plane values of non‐contact or indirect contact ACL injuries.^a^

Variable	Noncontact and indirect contact situational patterns					
	Total (*n* = 114)	Pressing/Tackling (*n* = 60)	Being tackled (*n* = 17)	Regaining balance after kicking (*n* = 8)	Landing from jumping (*n* = 6)	Other (*n* = 23)
Trunk rotation at IC						
Toward injured	5 (4%)	1 (2%)	1 (6%)	1 (12%)	0 (0)	2 (9%)
Neutral	35 (31%)	21 (35%)	5 (29%)	2 (25%)	3 (50%)	4 (17%)
Toward uninjured	43 (38%)	23 (38%)	7 (41%)	3 (38%)	2 (33%)	8 (35%)
Unsure	31 (27%)	15 (25%)	4 (24%)	2 (25%)	1 (17%)	9 (39%)
Trunk rotation at IF						
Toward injured	6 (5%)	1 (2%)	2 (12%)	1 (12%)	1 (17%)	1 (4%)
Neutral	8 (7%)	7 (12%)	0 (0)	0 (0)	0 (0)	1 (4%)
Toward uninjured	70 (61%)	39 (65%)	11 (65%)	5 (62%)	4 (67%)	11 (48%)
Unsure	30 (26%)	13 (22%)	4 (24%)	2 (25%)	1 (17%)	9 (39%)
Frontal plane hip alignment IC						
Abduction	75 (66%)	43 (72%)	11 (65%)	5 (62%)	3 (50%)	13 (57%)
Neutral	3 (3%)	1 (2%)	1 (6%)	0 (0)	0 (0)	1 (4%)
Adduction	1 (4%)	0 (0)	0 (0)	0 (0)	0 (0)	1 (4%)
Unsure	35 (31%)	18 (30%)	6 (35%)	3 (38%)	3 (50%)	9 (39%)
Frontal plane hip alignment IF						
Abduction	66 (58%)	38 (63%)	10 (59%)	5 (62%)	3 (50%)	10 (43%)
Neutral	6 (5%)	2 (3%)	1 (6%)	1 (12%)	0 (0)	0 (0)
Adduction	3 (3%)	2 (3%)	0 (0)	0 (0)	0 (0)	4 (17%)
Unsure	39 (34%)	18 (30%)	6 (35%)	3 (38%)	3 (50%)	9 (39%)
Frontal plane knee alignment IC						
Valgus	8 (7%)	5 (8%)	1 (6%)	0 (0)	0 (0)	2 (9%)
Neutral	53 (46%)	33 (55%)	7 (41%)	5 (62%)	0 (0)	8 (35%)
Varus	0 (0)	0 (0)	0 (0)	0 (0)	0 (0)	0 (0)
Unsure	53 (46%)	22 (37%)	9 (53%)	3 (38%)	6 (100%)	13 (57%)
Frontal plane knee alignment IF						
Valgus	62 (54%)	40 (67%)	7 (41%)	3 (38%)	1 (17%)	11 (48%)
Neutral	4 (4%)	1 (2%)	2 (12%)	1 (12%)	0 (0)	0 (0)
Varus	0 (0)	0 (0)	0 (0)	0 (0)	0 (0)	0 (0)
Unsure	48 (42%)	19 (32%)	8 (47%)	4 (50%)	5 (83%)	12 (52%)
Foot position at IC						
External	48 (42%)	31 (52%)	7 (41%)	5 (62%)	1 (17%)	4 (17%)
Neutral	13 (11%)	8 (13%)	2 (12%)	0 (0)	0 (0)	3 (13%)
Internal	0 (0)	0 (0)	0 (0)	0 (0)	0 (0)	0 (0)
Unsure	53 (46%)	21 (35%)	8 (47%)	3 (38%)	5 (83%)	16 (70%)
Foot position at IF						
External	53 (46%)	35 (58%)	8 (47%)	5 (62%)	1 (17%)	4 (17%)
Neutral	13 (11%)	8 (13%)	1 (6%)	0 (0)	0 (0)	4 (17%)
Internal	0 (0)	0 (0)	0 (0)	0 (0)	0 (0)	0 (0)
Unsure	48 (42%)	17 (28%)	8 (47%)	3 (38%)	5 (83%)	15 (65%)
Significant hip IR/ADD from IC to IF						
Yes	60 (53%)	38 (63%)	7 (41%)	3 (38%)	2 (33%)	10 (43%)
No	8 (7%)	4 (7%)	2 (12%)	1 (12%)	0 (0)	1 (4%)
Unsure	46 (40%)	18 (30%)	8 (47%)	4 (50%)	4 (67%)	12 (52%)
Significant increase in valgus alignment from IC to IF						
Yes	23 (20%)	14 (23%)	4 (24%)	0 (0)	0 (0)	5 (22%)
No	42 (37%)	24 (40%)	5 (29%)	5 (62%)	2 (33%)	6 (26%)
Unsure	49 (43%)	22 (37%)	8 (47%)	3 (38%)	4 (67%)	12 (52%)

Abbreviations: 2D, two‐dimensional; ACL, anterior cruciate ligament; ADD, adduction; IC, initial contact; IF, injury frame; IR, internal rotation.

^a^ Data are reported as *n* (%). Categorical observations are derived from 2D video footage and should be interpreted as visual estimates rather than precise biomechnical measurements.

### Contextual characteristics of indirect and non‐contact injuries

**Figure 7:**
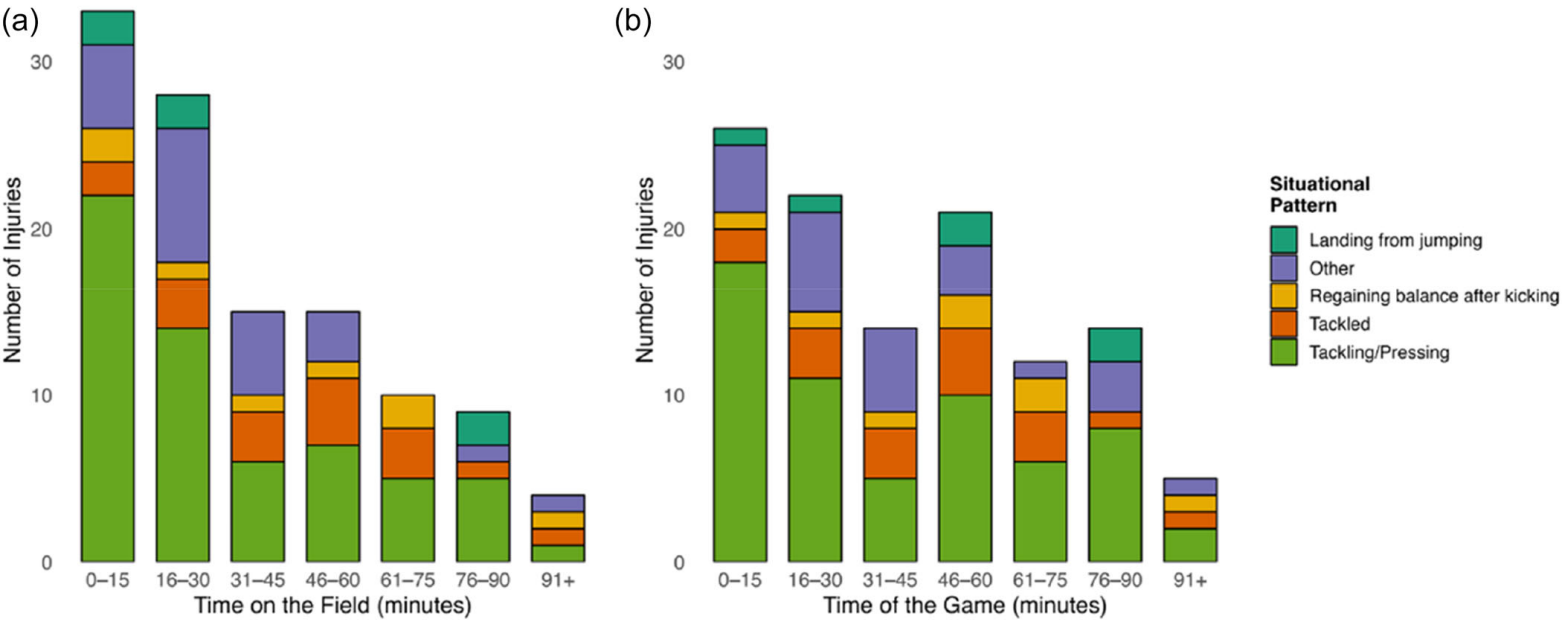
(a) Distribution of ACL injury situational patterns by time on the field. (b) Distribution of ACL injury situational patterns by time of the game. ACL, anterior cruciate ligament.

Contextual characteristics (Table 4) showed that ACL injuries sustained during pressing and tackling most frequently occurred on natural turf (69%), in the first half (62%) and during drawn match scenarios (58%). Overall, 37% occurred within the first 15 min of a player's time on the field (Figure 6a), and 30% within the first 15 min of the match play (Figure 6b). Non‐contact pressing injuries were evenly distributed across the 0–15 and 16–30 min intervals (30.8% each), whereas indirect contact pressing injuries occurred more often within the first 15 min (50%). For tackling, non‐contact cases peaked early (50% in 0–15 min), while indirect contact injuries occurred more often between 16–30 min (35.7%). When combined, 37% of pressing and tackling occurred within the first 15 min of a player's time on the field and 30% within the first 15 min of match play, with smaller proportions across later intervals (Figure 7a,b). No statistically significant associations were observed with match status (*χ* ^2^ = 0.135) or turf type (*χ* ^2^ = 0.351).

**Table 4:** Characteristics of all video ACL injuries and for noncontact and indirect contact injuries across identified situational patterns.^a^

Variable	All video injuries (*n* = 127)	Noncontact and indirect contact situational patterns					
		Pressing/Tackling	Being tackled	Regaining balance after kicking	Landing from jumping	Other	Total (*n* = 114)
Precipitation							
Yes	5 (4%)	2 (3%)	0 (0)	0 (0)	3 (13%)	0 (0)	5 (4%)
No	122 (96%)	58 (97%)	17 (100%)	8 (100%)	6 (100%)	20 (87%)	109 (96%)
Turf							
Artificial	35 (27%)	14 (24%)	4 (24%)	1 (12%)	3 (50%)	8 (35%)	30 (27%)
Natural	78 (62%)	41 (69%)	12 (71%)	7 (88%)	2 (33%)	10 (43%)	72 (64%)
Combination	13 (10%)	4 (7%)	1 (6%)	0 (0)	1 (17%)	5 (22%)	11 (10%)
Unsure	1 (1%)	0 (0)	0 (0)	0 (0)	0 (0)	0 (0)	0 (0)
Match entry status							
Starter	113 (89%)	52 (87%)	17 (100%)	7 (88%)	4 (67%)	20 (87%)	100 (88%)
Reserve	14 (11%)	8 (13%)	0 (0)	1 (12%)	2 (33%)	3 (13%)	14 (12%)
Match scoreline							
Winning	22 (17%)	11 (18%)	2 (12%)	3 (38%)	0 (0)	4 (18%)	20 (18%)
Losing	38 (30%)	14 (23%)	8 (47%)	1 (12%)	4 (67%)	9 (39%)	36 (32%)
Drawing	67 (53%)	35 (59%)	7 (41%)	4 (50%)	2 (33%)	10 (43%)	58 (50%)
Half of play							
First	73 (58%)	37 (62%)	8 (47%)	3 (38%)	2 (33%)	15 (65%)	65 (57%)
Second	54 (42%)	23 (38%)	9 (53%)	5 (62%)	4 (67%)	8 (35%)	49 (43%)
Time of the game							
0–15 min	30 (24%)	18 (30%)	2 (12%)	1 (12%)	1 (17%)	4 (17%)	26 (23%)
16–30 min	24 (19%)	12 (20%)	3 (12%)	1 (20%)	1 (17%)	6 (26%)	23 (20%)
31–45 min	19 (15%)	7 (12%)	3 (19%)	1 (12%)	0 (0)	5 (22%)	16 (14%)
46–60 min	22 (17%)	8 (13%)	5 (29%)	2 (25%)	2 (33%)	3 (13%)	20 (18%)
61–75 min	13 (10%)	5 (8%)	3 (19%)	2 (25%)	0 (0)	1 (4%)	11 (10%)
76–90 min	15 (11%)	8 (13%)	0 (0)	0 (0)	2 (33%)	4 (17%)	14 (12%)
90+min	4 (3%)	2 (4%)	1 (6%)	1 (12%)	0 (0)	0 (0)	4 (4%)
Time on the field							
0–15 min	38 (30%)	22 (37%)	2 (12%)	2 (25%)	2 (33%)	6 (26%)	34 (30%)
16–30 min	30 (24%)	16 (27%)	3 (18%)	1 (12%)	2 (33%)	7 (30%)	29 (25%)
31–45 min	17 (13%)	5 (8%)	3 (18%)	1 (12%)	0 (0)	5 (22%)	14 (12%)
46–60 min	16 (13%)	6 (10%)	4 (24%)	1 (12%)	0 (0)	3 (13%)	14 (12%)
61–75 min	12 (9%)	5 (8%)	3 (18%)	2 (25%)	0 (0)	0 (0)	10 (9%)
76–90 min	10 (8%)	5 (8%)	1 (6%)	0 (0)	2 (33%)	1 (4%)	9 (8%)
90+min	4 (3%)	1 (2%)	1 (6%)	1 (12%)	0 (0)	1 (4%)	4 (4%)
Position							
Forward	38 (30%)	16 (27%)	8 (47%)	5 (62%)	2 (33%)	4 (17%)	35 (31%)
Midfielder	42 (34%)	17 (28%)	6 (35%)	1 (12%)	0 (0)	12 (52%)	36 (32%)
Defender	41 (33%)	27 (45%)	3 (18%)	2 (25%)	0 (0)	5 (22%)	37 (32%)
Goalkeeper	6 (5%)	0 (0)	0 (0)	0 (0)	4 (67%)	2 (9%)	6 (5%)

Abbreviation: ACL, anterior cruciate ligament.

^a^ Data are reported as *n* (%).

Injuries sustained while being tackled (*n* = 17) occurred exclusively in starting players (100%), slightly more often in the second half (53%) and during losing scenarios (47%). These injuries were evenly distributed across match and playing periods, with a small peak between 46–60 min (24%–25%). No significant associations were observed with match status (*χ* ^2 ^= 0.346) or turf type (*χ* ^2 ^= 0.925).

Regaining balance after kicking (*n* = 8) occurred most frequently on natural turf (88%), in the second half (62%) and during drawn matches (50%). The most common injury windows were the first 15 min and 61–75 min (25% each). There were no significant associations with match status (*χ* ^2^ = 0.211) or turf type (*χ* ^2^ = 0.438).

Landing from jumping injuries (*n* = 6) primarily involved goalkeepers (67%), occurred more often in the second half (67%) and during losing scenarios (67%). No inferential statistical testing was performed for this group due to the small sample size.

## DISCUSSION

The purpose of this study was to describe the situational and biomechanical characteristics of ACL injuries in professional women's football using a structured video analysis approach. Four key findings emerged: (1) 90% of ACL injuries occur without direct contact to the knee; (2) defensive actions, particularly pressing or tackling, were the most common scenarios (53%); (3) joint and trunk kinematics varied across different situational patterns, with observations derived from smaller biomechanical subsamples (57 frontal/transverse and 46 sagittal cases) and (4) injuries frequently occur early in matches, during drawn scorelines. These findings highlight common scenarios and movement patterns that can inform targeted injury prevention strategies, rehabilitation planning, return to sport and future research in women's football.

### Mechanism of injury

Consistent with prior literature, our findings confirmed that most ACL injuries in women's football are non‐contact or indirect contact in nature (90%), consistent with reports from women's (84%–88%) [1, 32] and men's football (84%–88%) [19, 20, 45]. Similar patterns are observed in other elite women's sports such as basketball (44% non‐contact, 56% indirect contact), Australian football (54% non‐contact, 46% indirect) and netball (50% non‐contact, 50% indirect) [27, 41, 43]. In our study, 65% of injuries occurred during defensive actions (pressing/tackling), similar to Australian football, where injuries typically result from defensive cutting [41]. In contrast, basketball and netball show comparable cutting or landing mechanisms, but these typically arise in an offensive situation [27, 43].

### Situational pattern and football context

Defensive situations had the highest prevalence of ACL injuries (65%), consistent with previous findings in professional women's football (63%–69%) [32, 34] and similar to rates reported in the men's game (65%) [14, 19, 20, 45]. The most frequent situational pattern was pressing and tackling (53%), typically occurring in ground‐based duels characterised by high horizontal velocity and low vertical displacement. These findings echo prior studies in both sexes and reinforce that pressing actions are common situational patterns in ACL injury [1, 14, 19, 20, 32, 34]. Injury frequency was highest in the first half (61%) and when the score was tied (58%), with nearly three‐quarters (72%) occurring within the first 45 min of a player's time on the field. These early phases of matches are typically characterised by elevated tactical urgency and physical output. High‐speed running, accelerations and decelerations can be up to 25% higher in the first 15 min compared to the final 15 min of play [6], reflecting teams' efforts to assert early dominance. Pressing actions are more frequent in teams that go on to win [36], and with ~75% of matches won by the team that scores first [42], early pressing becomes a key tactical priority. This context may help explain why a substantial proportion of ACL injuries occur during early‐match defensive actions, when elevated physical demands and cognitive load converge to challenge neuromuscular control and decision making. These patterns may also coincide with periods of acute or transient fatigue, from high‐intensity efforts or the cumulative effects of inadequate load management across matches, travel and training.

Being tackled (15%) was the second most common situational pattern and the most frequent offensive scenario in this cohort. This proportion is slightly higher than previously reported rates in women's football (8%–13%) [1, 32]; however, lower than a more recent analysis (25%) [34], and falls just below the range reported in the men's game (18%–23%) [14, 19, 20, 45]. A large proportion of these injuries were indirect (82%) and involved upper body contact (67%), indicating that many of these events occurred during contested duels rather than isolated, unopposed movements. Notably, none of these injuries occurred in the defensive third, despite many being sustained by defenders (45%), perhaps reflecting the evolving modern tactical roles that demand greater involvement in advanced areas of the pitch, such as overlapping runs, pressing or transitional duels in the midfield and attacking third [11].

Regaining balance injuries (7%) were notably less frequent in this cohort compared to prior reports in professional women's football (22%) [1, 32], and more closely resemble rates observed in recent literature in women's (8%) [34] and men's football (6%–15%) [14, 19, 20, 45]. This discrepancy from earlier literature may reflect several converging factors: a broader and more diverse sample spanning multiple leagues and playing levels, potential differences in tactical styles and positional roles and the increasing implementation of targeted neuromuscular training and injury prevention programs that may have mitigated certain high‐risk postural loss scenarios, though further investigation is warranted. The incidence of landing‐related injuries (6%) was consistent with both women's (3%–11%) and men's (6%–11%) literature [1, 14, 19, 20, 32, 34, 45]. Most of these injuries involved goalkeepers (67%) and were characterised by high vertical velocity (83%), reflecting a position‐specific mechanism rather than a generalised match context such as an aerial duel. This highlights the unique loading patterns and movement demands placed on goalkeepers, warranting tailored prevention strategies for this role.

### Biomechanics

The biomechanical profiles captured in this study are broadly consistent with previous video analysis reporting relatively limited flexion at the trunk, hip and knee, accompanied by ipsilateral trunk flexion, rotation toward the uninjured side, hip abduction and external foot rotation, progressing toward hip internal rotation and adduction at IF [32, 34]. However, whereas Lucarno et al. reported heel strike at IC in 71% of cases and Iborra et al. observed a higher prevalence of flat‐foot contact (53%), our cohort demonstrated a more even distribution between heel and flat foot strikes (32% and 28%, respectively), although 40% of cases were classified as unsure due to video clarity. These differences may partly reflect variation in playing surface, footwear, competition level or the broader range of leagues represented in the present cohort. However, they may also arise from methodological differences, including variation in video quality and case classification, and therefore should be interpreted cautiously as true biomechanical differences between studies.

In pressing/tackling scenarios, players displayed a neutral trunk angle (~0°) with ipsilateral side flexion, moderate hip flexion (~50°) and limited knee flexion at IC (~24°), increasing to ~60° at IF. Among assessable cases, flat‐foot positioning (69%), valgus knee alignment (67%, though 32%–37% of knee alignment observations were unsure) and hip internal rotation/adduction (63%) were commonly observed. These sagittal and transverse plane kinematics broadly resemble those reported in men's football [14, 19, 20]. Notably, our cohort demonstrated greater knee flexion at IF (~60°) than male professional English football players (37.5°; [20]) and a more even distribution of foot strike at IC (heel: 37%; flat: 35%), compared with the heel‐dominant profiles typically reported in men (57%–69%) [14, 19], although Della Villa et al. reported a higher rate of flat foot strikes (67%) [20]. These differences may reflect sex‐based movement strategies, situational constraints during the injury event or variability in how foot strike is defined across studies.

By contrast, players injured while being tackled exhibited greater trunk tilt (15°–35° at IF), likely reflecting indirect upper‐body contact, with a modest increase in trunk flexion (10°–15°). Among assessable cases, valgus knee alignment was frequently observed at IF (~85%), consistent with male data (59%–85%) [20], although valgus collapse rates should be interpreted cautiously given the small assessable sample (*n* = 9). Hip flexion (55°–60°) and knee flexion changes (IC to IF) were broadly comparable to Buckthorpe et al. [14], while knee flexion changes appeared greater than those reported by Della Villa et al. (15°–25°) [20]. Shared features included trunk rotation toward the uninjured side, hip abduction and flat foot strikes. Collectively, these preliminary observations tentatively suggest that women may exhibit slightly deeper joint flexion at IF, with the overall kinematic sequence during tackling injuries appearing broadly similar to that of male cohorts; however, given the small assessable sample, this should be considered hypothesis‐generating and warrants investigation in larger cohorts.

In balance‐recovery scenarios following a kick, players demonstrated an upright posture (−10° at IC, −5° at IF), minimal changes in hip flexion (10°–30°) and increased knee flexion (45°–60°). This more upright posture, with limited sagittal plane engagement, differs from male cohorts, who tend to exhibit greater flexion at these joints [14, 19, 20], potentially altering load distribution and risk mechanisms. Landing‐related injuries, often observed in goalkeepers (67%), showed similar upright trunk posture (0° at both IC and IF), moderate knee flexion increases (40°–60°) and low hip flexion (10°–30°), differing from 5° to 30° trunk flexion seen in the male players [14, 19, 20]. These kinematic patterns may limit force attenuation and increase anterior tibial shear, though findings are drawn from a very small assessable sample (*n* = 3) for sagittal analysis) and should be considered hypothesis‐generating. They nonetheless highlight the value of investigating goalkeeper‐specific neuromuscular training targeting landing mechanics.

### Implications and future research

This study addresses two critical gaps in the literature on ACL injuries in women's football: the contextual factors surrounding injury events and the biomechanics associated with common situational patterns. The concentration of injuries during early‐match defensive actions (particularly pressing and tackling) highlights a period where risk may be elevated, potentially driven by transient neuromuscular fatigue, cognitive load and acute match intensity. These findings suggest that existing warm‐up protocols and early match preparation strategies may need revision to mitigate vulnerability during this critical phase.

Preventative programs such as the 11+ (formally FIFA11+) have demonstrated efficacy in reducing non‐contact ACL injuries [16]. Our findings support enhancing these programs by incorporating scenario‐specific neuromuscular training, tailored to playing positions. For example, goalkeepers might benefit from targeted interventions to improve landing mechanics and vertical load attenuation as they sustain the highest number of landing‐related injuries. Similarly, midfielders and defenders may require greater exposure to high‐intensity pressing and tackling tasks to reinforce safe movement strategies and improve decision‐making under pressure. They could be integrated into the warm‐up component of the 11+, without significant time disruption. In the rehabilitation context, incorporating situational pattern training during late‐stage recovery may optimise return‐to‐play outcomes. Reintroducing match‐relevant tasks that simulate chaotic and cognitively demanding gameplay aligns with emerging recommendations to ensure physical readiness and psychological resilience [15, 44].

This study highlights the need to examine how transient neuromuscular fatigue and cumulative load contribute to ACL injury risk, particularly during early‐match defensive actions such as pressing and tackling. The high incidence of injuries in these periods suggests compromised movement control under elevated physical and cognitive demands. Future research should explore proximal control strategies and whole‐body coordination, especially in scenarios involving upper‐body perturbations, such as being tackled. The frequent involvement of goalkeepers in landing‐related injuries also supports the investigation of position‐specific mechanisms and neuromuscular strategies. Further work is needed to evaluate reactive, role‐relevant biomechanical assessments and the effectiveness of context‐specific prevention and rehabilitation drills, including dynamic pressing and landing tasks. Such studies will help refine injury mitigation approaches tailored to the demands of women's football.

## LIMITATIONS

Several limitations warrant consideration. First, variability in video quality and camera angles limited the number of cases eligible for biomechanical analysis, reducing the precision of joint angle assessments and resulting in a high proportion of ‘unsure’ classifications. Consequently, differences across scenarios should therefore be considered hypothesis‐generating rather than definitive. Although a validated video analysis protocol was employed, the use of 2D broadcast footage is inherently less precise than gold‐standard approaches such as model‐based image matching or three‐dimensional (3D) motion capture [48]. Second, the classification of situational patterns, particularly ‘being tackled’, may overlap with other actions, introducing potential misclassification [1]. Third, only match injuries were analysed; ACL injuries occurring during training may involve different mechanisms. Fourth, the absence of player‐specific data (e.g., anthropometrics, training/match load or injury history) precluded the examination of individual risk modifiers, and broad positional grouping may mask role‐specific biomechanical differences [9, 10]. Fifth, injury identification relied on publicly available sources without clinical MRI confirmation, preventing verification of injury severity, concurrent structural damage or precise diagnosis. Media‐based reporting may also introduce selection bias, as injuries involving prominent players or high‐profile matches are more likely to be reported, which should be considered alongside the study's multicentre design. Finally, because exposure data were unavailable, contextual proportions reflect injury distribution only and cannot be interpreted as incidence rates or relative risks.

## CONCLUSION

This study characterises the contextual and biomechanical features of ACL injuries in professional women's football, using a large, diverse sample across multiple leagues. Pressing and tackling, particularly early in a player's time on the field, were the most frequently observed scenarios, potentially reflecting tactical urgency, elevated physical demands and transient neuromuscular fatigue. Broadly consistent biomechanical profiles were observed when compared with the previous literature, though these findings are constrained by the limitations of 2D video analysis, small kinematic sub‐samples and high ‘unsure’ classification rates. While the study is limited by the descriptive nature of video‐based analysis and the absence of individual‐level risk factors, the findings provide descriptive insights into common injury scenarios in women's football and support the need for situation‐specific injury prevention strategies, while highlighting the priorities for further research into fatigue‐related mechanisms, contextual influences on injury risk and situation‐specific biomechanical patterns.

## AUTHOR CONTRIBUTIONS

Wyatt Hampstead conceived the study, led the study design and was the primary author responsible for drafting the manuscript. Ric Lovell, Matthew Whalan and Evangelos Pappas contributed to the conceptualisation of the study, refinement of the methodology and critical revision of the manuscript. Bradley Wakefield performed the statistical analyses and contributed to data interpretation and manuscript editing. Joshua Bonney and Lionel Chia were responsible for data collection, data analysis and manuscript editing. Vanessa Crespin contributed to data collection and provided substantive editorial input on the manuscript.

## FUNDING INFORMATION

The authors have no funding to report.

## CONFLICT OF INTEREST STATEMENT

The authors declare no conflicts of interest.

## ETHICS STATEMENT

Exemption was granted by the Human Research Ethics Committee at the University of Wollongong (HREC reference: 2024/316).

## Supporting information

Supplementary Material.

## Data Availability

The datasets used in this study were compiled from publicly accessible online sources. All materials are available to the public, and additional information regarding data retrieval can be supplied by the corresponding author upon request.
