# Supplementary material for: Mechanisms and situational patterns of anterior cruciate ligament injuries in professional women's football (soccer): A systematic video analysis across 25 leagues (2022–2024)
Source: Knee Surg Sports Traumatol Arthrosc. 2026 Jul 13;34(9):3441–58. doi: 10.1002/ksa.70528 (PMC13502420; doi:10.1002/ksa.70528)
Supplement: Supplementary file 1 [file KSA-34-3441-s001.docx]

**Supplementary Material**

**Supplementary Table 1:** List of leagues

| **League** | **Total** |
| --- | --- |
| **International** | 24 |
| **WSL** | 17 |
| **NWSL** | 15 |
| **D1 Arkema** | 9 |
| **SWPL 1** | 9 |
| **Serie A** | 7 |
| **Liga F** | 6 |
| **Liga MX** | 6 |
| **A-League Women** | 5 |
| **Bundesliga** | 4 |
| **Liga BPI** | 4 |
| **Brasileirão Feminino** | 3 |
| **Primera División A** | 3 |
| **Eredivisie Vrouwen** | 2 |
| **Toppserien** | 2 |
| **Arkema Première Ligue** | 1 |
| **Cypriot First Division** | 1 |
| **Damallsvenskan** | 1 |
| **Ekstraliga** | 1 |
| **Kadın Futbol Süper Ligi** | 1 |
| **Liga Femenina** | 1 |
| **Nationalliga A** | 1 |
| **Primera División** | 1 |
| **Super League** | 1 |
| **Women's Championship** | 1 |
| **Women's Super League** | 1 |
|  |  |

**Supplementary Table 2:** Checklist for video evaluation.

| **Variables** | **Categories** |
| --- | --- |
| Playing phase before injury | Defensive/Offensive |
| Injury Side | Right/Left |
| Long axis field location | Defensive third/Midfield third/Offensive third |
| short axis field location | Left side corridor/Middle corridor/Right side corridor |
| Player situational pattern (only for indirect and non-contact injuries | Pressing/tackling/being tackled/landing from jump/regaining balance after kicking, |
| Player contact preceding injury | Yes/No |
| If contact, where? | Upper body/Pelvis/Injured leg/Un-injured leg |
| Injury classification | Direct contact/Indirect contact/Non-contact |
| Leg loading at IF | Injured Leg/Un-injured Leg/None/Unsure |
| Horizontal speed | Zero/Low/High |
| Vertical speed | Zero/Low/High |

**Supplementary Table 3:** Checklist for biomechanical evaluation.

| **Variables** | **Categories** |
| --- | --- |
| Trunk flexion (+ flexion, - extension) | Estimation to nearest 5° |
| Hip flexion (+ flexion, - extension) | Estimation to nearest 5° |
| Knee flexion (+ flexion, - extension) | Estimation to nearest 5° |
| Ankle flexion (+ dorsi-flexion, - plantarflexion) | Estimation to nearest 5° |
| Foot strike | Heel/flat/toe/unsure |
| Trunk tilt (+ ipsilateral, - contralateral) | Estimation to nearest 5° |
| Trunk rotation | Towards injured leg/neutral/towards uninjured  leg/unsure |
| Frontal plane hip alignment | Abducted/neutral/adducted/unsure |
| Frontal plane knee alignment | Valgus/neutral/varus/unsure |
| Foot position | Externally rotated/neutral/internally rotated/unsure |
| Significant Hip IR/ADD from IC to IF? | Yes/no/unsure |
| Valgus collapse | Yes/no/unsure |

**Supplementary Table 4:** Checklist for contextual charateristics.

| **Variable** | **Category** |
| --- | --- |
| Precipitation | Yes/No |
| Turf | Artificial/Natural/ Combination/Unsure |
| Starter or Reserve | Starter/Reserve |
| Winning, losing or drawing | Winning/Losing/Drawing |
| Half of play | First/Second |
| Time of Game | 0-15/16-30/31-45/46-60/61-75/76-90/90+ |
| Time on the field | 0-15/16-30/31-45/46-60/61-75/76-90/90+ |
| Position | Forward/Midfield/Defender/Goalkeeper |

| **Variable** |  | **Noncontact and Indirect Contact Situational Patterns (n= 114)** | | | | | |
| --- | --- | --- | --- | --- | --- | --- | --- |
|  | All video injuries (n=127) | Pressing/  Tackling  (n=60) | Being Tackled  (n=17) | Regaining Balance After Kicking  (n=8) | Landing From Jumping  (n=6) | Other  (n=23) | Total (n=114) |
| Long Axis of the Field | | | | | | | |
| Defensive third | 33 (27%) | 16 (27%) | 0 (0) | 2 (25%) | 4 (67%) | 9 (41%) | 31 (28%) |
| Midfield third | 55 (45%) | 32 (54%) | 8 (50%) | 2 (25%) | 1 (17%) | 7 (32%) | 50 (45%) |
| Offensive third | 36 (29%) | 11 (19%) | 8 (50%) | 4 (50%) | 1 (17%) | 6 (27%) | 30 (27%) |
| Short Axis of the field | | | | | | | |
| Left side corridor | 38 (33%) | 18 (31%) | 6 (38%) | 1 (12%) | 0 (0) | 7 (32%) | 32 (29%) |
| Middle corridor | 45 (39%) | 17 (29%) | 3 (19%) | 5 (62%) | 4 (67%) | 10 (45%) | 39 (35%) |
| Right side corridor | 41 (36%) | 24 (41%) | 7 (44%) | 2 (25%) | 2 (33%) | 5 (23%) | 40 (36%) |
| Uncertain | 3 (3%) | 0 (0) | 0 (0) | 0 (0) | 0 (0) | 0 (0) | 0 (0) |
